# Asymmetric Biomechanical Properties of the Paravertebral Muscle in Elderly Patients With Unilateral Chronic Low Back Pain: A Preliminary Study

**DOI:** 10.3389/fbioe.2022.814099

**Published:** 2022-02-10

**Authors:** Zugui Wu, Xiangling Ye, Zixuan Ye, Kunhao Hong, Zehua Chen, Yi Wang, Congcong Li, Junyi Li, Jinyou Huang, Yue Zhu, Yanyan Lu, Wengang Liu, Xuemeng Xu

**Affiliations:** ^1^ The Fifth Clinical Medical College, Guangzhou University of Chinese Medicine, Guangzhou, China; ^2^ Guangdong Second Traditional Chinese Medicine Hospital, Guangzhou, China; ^3^ Baishui Health Center, Qujing, China; ^4^ Luoyang Orthopedic Hospital Of Henan Province (Orthopedic Hospital of Henan Province), Zhengzhou, China

**Keywords:** biomechanical properties, paravertebral muscle, muscle tone, stiffness, chronic low back pain

## Abstract

**Background:** Clinical incidences of chronic low back pain among the elderly are increasing. However, studies have not fully elucidated on changes in biomechanical properties of paravertebral muscles in patients with unilateral chronic low back pain. We evaluated the changes in biomechanical properties of painful and non-painful paravertebral muscles in elderly patients with unilateral chronic low back pain.

**Methods:** Biomechanical properties of paravertebral muscles, including muscle tone and stiffness, in elderly patients with unilateral chronic low back pain were measured using MyotonPRO. Lumbar Lordosis and Sacral Slope were measured by magnetic resonance imaging. Cross-sectional areas of paravertebral muscles were evaluated using ImageJ software version 1.53. Chronic low back pain severity was assessed by Visual Analogue Scale (VAS) and Oswestry Disability Index (ODI) scores. The correlations between VAS scores, ODI scores, Lumbar Lordosis, Sacral Slope, cross-sectional areas (painful side), disease duration, and biomechanical properties of paravertebral muscles in the painful side were analyzed.

**Results:** A total of 60 elderly patients with unilateral chronic low back pain were enrolled in this study. The muscle tone and stiffness of paravertebral muscles on the painful side were significantly higher than those on the non-painful side (*p* < .05). Cross-sectional areas of paravertebral muscles on the painful side at the L3 level were smaller than those of the non-painful side (*p* < .05). The VAS scores and ODI scores were significantly positively correlated with muscle tone and stiffness of paravertebral muscles on the painful side (*p* < .05 and *p* < .01, respectively). There were no significant correlations between disease duration, cross-sectional areas (painful side), Lumbar Lordosis, or Sacral Slope and muscle tone and stiffness of paravertebral muscles on the painful side (*p* > .05).

**Conclusion:** In elderly patients with unilateral chronic low back pain, muscle tone and stiffness of paravertebral muscles on the painful side are higher than for those on the non-painful side. The asymmetry of biomechanical properties of paravertebral muscles is associated with severity of chronic low back pain.

## Introduction

Chronic low back pain (CLBP) is one of the leading causes of disability in the elderly (Vos et al., 2012; Morlion, 2013; GBD 2016 Disease and Injury Incidence and Prevalence Collaborators, 2017). It is defined as pain that occurs in the area from the lower back edge of the ribs to the upper edge of gluteal muscles for more than 3 months (Airaksinen et al., 2006). The pain and dysfunction caused by CLBP affect patients’ quality of life and often require medical attention, which significantly increases healthcare costs (Ma et al., 2014). At the same time, chronic low back pain is also a common cause of delayed working hours, and the economic loss caused by delayed working hours is huge (Guo et al., 1999). The increase in medical cost and the loss of working time, these factors impose a significant socio-economic burden on patients (Guo et al., 1999; Freburger et al., 2009; Ma et al., 2014; Foster et al., 2018). Biomechanical characteristics include muscle tone, stiffness, Lumbar Lordosis, Sacral Slope, etc., which are closely related to the occurrence of Lumbar and back diseases (Chaléat-Valayer et al., 2011; Andonian et al., 2015). The Sacral Slope (sagittal) was defined as the angle between the upper edge of the sacral endplate and the horizontal line. There are many evaluation methods for Sacral Slope, and the most commonly used is the four-line Cobb method. Muscle tone is the inherent pressure of a muscle when it does not contract spontaneously. Stiffness indicates the ability of a muscle to resist contraction as a result of external forces (the pressure to deform it). In addition, biomechanical properties of muscles, such as muscle tone and stiffness, form the basis for maintaining muscle functionality and are critical for maintaining body balance as well as postural stability (Masi and Hannon, 2008). Biomechanical properties of lower back muscles affect the stability of the spine (White et al., 2018). Patients with CLBP have been shown to exhibit altered muscle tone and stiffness of paravertebral muscles (Haładaj and Topol, 2016; Nair et al., 2016), which may be associated with symptoms of CLBP and underlying pathological mechanisms (Kawchuk et al., 2001). Pain increases muscle tone, which then aggravates pain, leading to improper postural control (Roland, 1986; Lo et al., 2019). Although there are many methods for treating CLBP (Rubinstein et al., 2011; Haładaj and Topol, 2016), selection of appropriate intervention measures and their effects should be clinically assessed (Kravitz et al., 1981; Shah et al., 2020). Manual palpation is often used for clinical evaluation of musculoskeletal diseases, such as lower back muscles and spine (Abbott et al., 2009; Masaki et al., 2017). However, the reliability and stability of manual palpation has not been ascertained (Seffinger et al., 2004; Jonsson and Rasmussen-Barr, 2018). MyotonPRO is a non-invasive muscle monitoring device that can quickly measure the biomechanical properties of muscles, such as muscle tone and stiffness (Aird et al., 2012; Bailey, 2013). It is highly accurate when applied to healthy people or patients (Hu et al., 2018; Lohr et al., 2018).

Differences in muscle tone and stiffness of paravertebral muscles between young patients with CLBP and healthy people have been reported (Ilahi et al., 2020). However, the study did not compare nor investigate whether there are differences on both sides (left and right) of paravertebral muscles. Although CLBP incidences in young people are gradually increasing, they are more prevalent among the elderly. However, only a limited number of studies have evaluated the biomechanical properties of paravertebral muscles in elderly CLPB patients. Previously, we reported on differences in tone and stiffness of paravertebral muscles between elderly CLBP patients and healthy people (Wu et al., 2020). Our results showed that the tone and stiffness of paravertebral muscles in patients with bilateral CLBP were significantly higher than those in healthy people. However, the elasticity of paravertebral muscles in patients with bilateral CLBP was significantly lower than that in healthy people. In addition, the study also found that the changes in biomechanical properties were related to the degree of pain. But, we only included patients with bilateral CLBP. In patients with unilateral CLBP, the non-painful side can be used as a control for the painful side (Wand et al., 2014). It is, therefore, imperative to evaluate the symmetry of muscles on both sides of the spine as this may provide critical information for clinical treatment. Liu et al. (2019) found that, when compared to the convex side, muscle tone and stiffness were significantly higher on the concave side of paravertebral muscles in adolescent patients with idiopathic scoliosis. Other studies have also reported significant differences in cross-sectional areas of paravertebral muscles between painful and non-painful sides of patients with unilateral CLBP, and that paravertebral muscle asymmetry is significantly related to CLBP (Barker et al., 2004). Therefore, there is a close association between lower back diseases and asymmetric changes in paravertebral muscles. However, studies have not evaluated the differences in biomechanical properties of paravertebral muscles between painful and non-painful sides in elderly patients with unilateral CLBP.

In this study, we used Magnetic resonance imaging (MRI) technology to measure the participants’ Lumbar Lordosis, Sacral Slope and Cross-Sectional Areas of paravertebral muscles, and MyotonPRO to measure the muscle tone and stiffness of the paravertebral muscles. We compared the muscle tone and stiffness of paravertebral muscles in the painful and non-painful sides of elderly patients with unilateral CLBP. In addition, correlations between Visual Analogue Scale (VAS) scores, Oswestry Disability Index (ODI) scores, disease duration, cross-sectional areas (CSAs) (painful side), Lumbar Lordosis, and Sacral Slope as well as biomechanical parameters of the painful paravertebral muscles were investigated. Our findings elucidate on the pathogenesis of CLBP and inform on its prevention and treatment.

## Methods

### Study Participants

Sixty (30 males and 30 females) elderly patients, aged between 60 and 75 years, with unilateral CLBP were recruited in this study. Their data were obtained from the orthopedic clinic of Guangdong Second Traditional Chinese Medicine Hospital. The inclusion criteria were (GBD 2016 Disease and Injury Incidence and Prevalence Collaborators, 2017): Aged 60–75 years (Morlion, 2013); Low back pain symptoms lasting more than 12 weeks (below the 12 thoracic vertebrae to the upper edge of the buttocks) (Vos et al., 2012); Unilateral CLBP (left and right sides are not restricted) (Airaksinen et al., 2006); No history of spinal surgery and (Ma et al., 2014) No intervention at least 4 weeks prior to inclusion in this study. The exclusion criteria were (GBD 2016 Disease and Injury Incidence and Prevalence Collaborators, 2017): Body mass index (BMI) ≥ 30 kg/m^2^ (Morlion, 2013); Presence of other diseases related to low back pain, including scoliosis, ankylosing spondylitis, and other related diseases (Vos et al., 2012); A history of spinal surgery (Airaksinen et al., 2006); The presence of neurological diseases, such as Parkinson’s disease among others. The research team screened and recruited eligible participants and obtained their relevant clinical information, including name, gender, age, height, weight, and information on the history of CLBP, such as disease duration and painful side among others. The research members involved in collecting basic and CLBP information for the participants were not involved in assessment of biomechanical parameters and cross-sectional areas of paravertebral muscles.

### Assessment of Biomechanical Properties of the Paravertebral Muscles

The research team member that performed this assessment had been professionally trained by Myoton and passed the professional assessment test. This researcher had more than 3 years of experience operating MyotonPRO. MyotonPRO (MyotonPRO^®^, Estonia) was used to measure biomechanical properties of paravertebral muscles in the orthopedic outpatient department of Guangdong Second Traditional Chinese Medicine Hospital. Before tests were performed, study participants were asked to rest on beds for 10 min to relax their muscles (Andonian et al., 2015). At the start of the test, participants were instructed to lie in prone positions with their heads in the center of the examination bed, and with their hands on both sides of the body. Before commencing the test, upper edges of the iliac spine on both sides were first palpated, the level between L3 and L4 spinous processes were determined, and then, the position between L1 to L5 spinous processes were identified and marked. The bulge of paravertebral muscles on both sides of the marked point served as the assessment site (Nair et al., 2016; Hu et al., 2018; Lo et al., 2019). The assessment begun from L1 to L5 on the left side and then on the right side. To reduce the interference of abdominal pressure fluctuations, participants were asked to hold their breath for 5 s at the end of expiration, before performing the tests (Hu et al., 2018). Two members of the research team performed the tests on a single participant, and the average of the two measurements was taken as the final value. The coefficient of variation was observed after each measurement, and if greater than 3%, the patient was reassessed. Measurements taken by MyotonPRO included those for muscle tone and stiffness.

### Parameters Used in MyotonPRO

Muscle tone and stiffness of bilateral paravertebral muscles were measured at each level from L1 to L5. The MyotonPRO probe was vertically mounted on the surface of measuring mark point, and then pressed to the appropriate depth, which was indicated by a change in light color from red to green. The device sent a mechanical pulse through the probe that stimulated the muscle, resulting in natural damping oscillations. Based on the natural damped oscillation signals, the MyotonPRO equipment automatically calculated muscle tone and stiffness. Muscle tone (F, measured in Hz) is the inherent pressure of a muscle when it does not contract spontaneously. The higher the Hz value, the higher the muscle tone (Van Deun et al., 2018). Stiffness (S, measured in N/m) indicates the ability of a muscle to resist contraction as a result of external forces (the pressure to deform it). The higher the N/m value, the higher the stiffness (Schneider et al., 2015).

### Measurement of Lumbar Lordosis, Sacral Slope and Cross-Sectional Areas of Paravertebral Muscles

**FIGURE 1:**
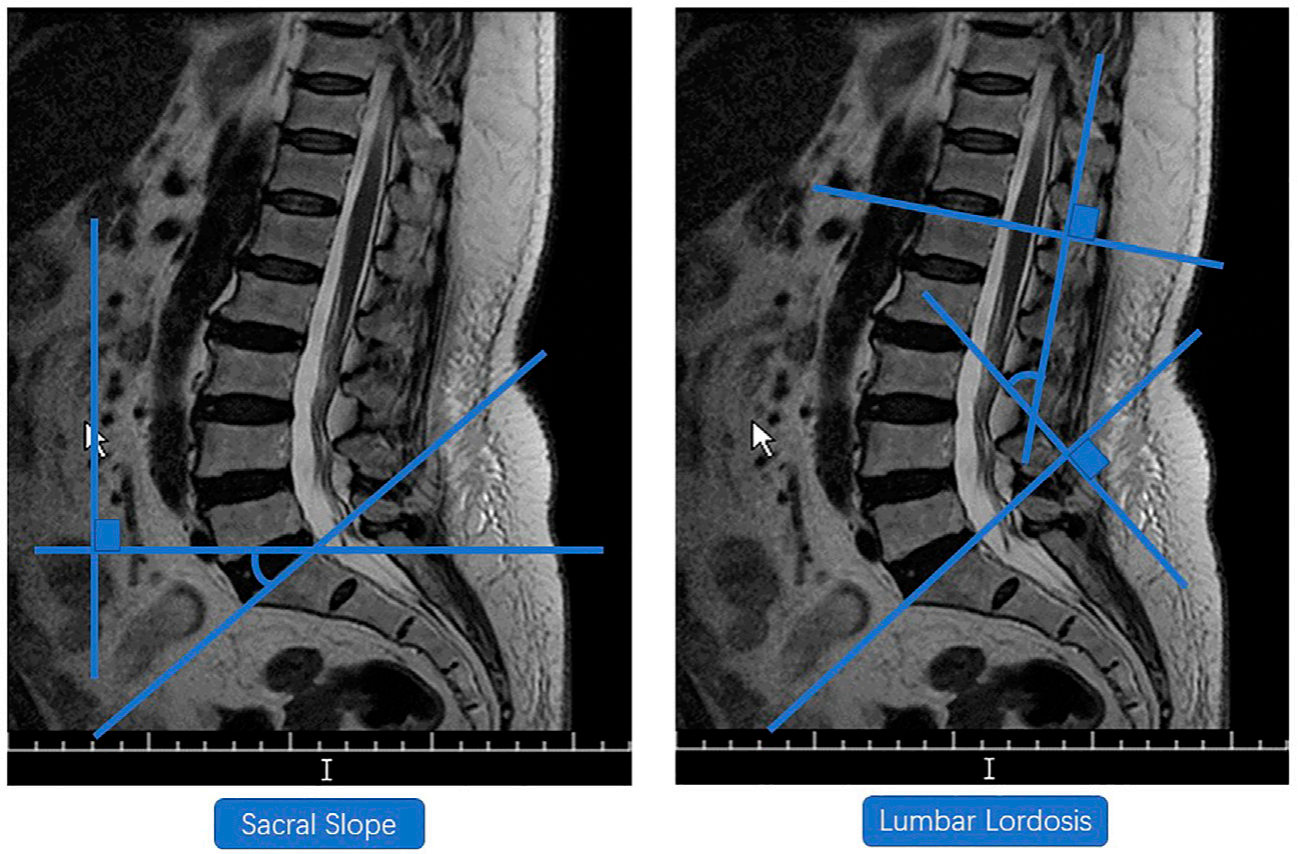
Lumbar lordosis and sacral slope.

**FIGURE 2:**
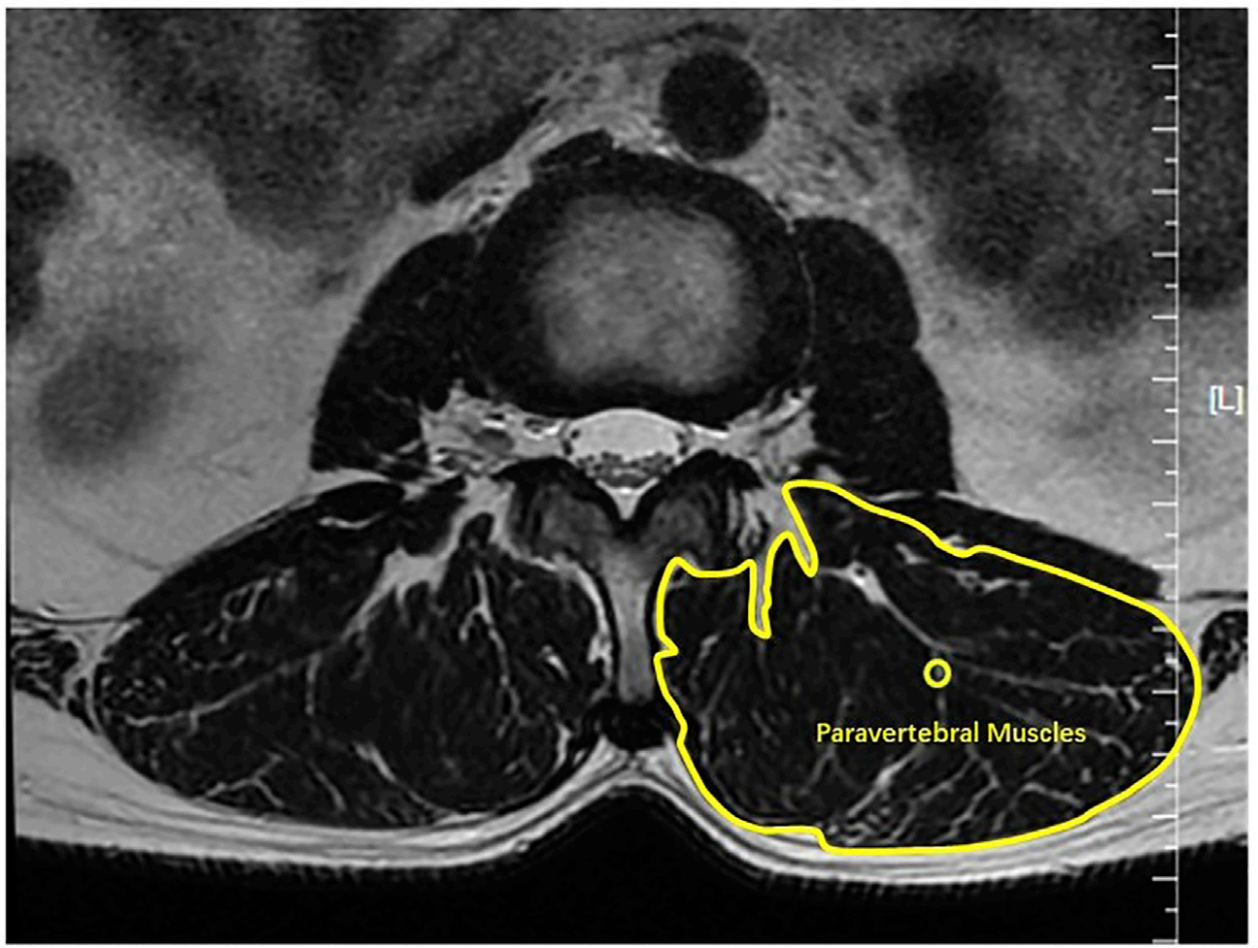
The cross-sectional area of the paravertebral muscles.

The Lumbar Lordosis, Sacral Slope, and the cross-sectional areas of paravertebral muscles were measured using the Signa HDx 1.5-T MRI system (GE Healthcare, Milwaukee, WI, United States). The Signa HDx 1.5-T MRI system with a spine coil was used to obtain T2 weighted sagittal and transverse MR images: T2 weighted sagittal [repetition time (TR), 4,000 ms; echo time (TE), 100 ms; field of view (FOV), 30 cm; matrix, 320 × 256; slice thickness, 4 mm; interslice gap, 1 mm; number of excitations (NEX), 2]; T2 weighted transverse [repetition time (TR), 5,100 ms; echo time (TE), 85 ms; field of view (FOV), 16 cm; matrix, 256 × 192; slice thickness, 4 mm; interslice gap, 1 mm; number of excitations (NEX), 2)]. Single-voxel point-resolved spectroscopy sequence was set with parameters as follows: repetition time (TR), 2000 ms; echo time (TE), 35 ms; average number of signals, 64; voxel of interest (VOI), 15 × 15 × 15 mm^3^ (3.4 ml); and acquisition time, 164 s. The Lumbar Lordosis and Sacral Slope of all participants were measured using the MRI technology. Measurements of Lumbar Lordosis and Sacral Slope were performed by a radiologist with 10 years of experience. The Sacral Slope was defined as the angle between the upper edge of the sacral endplate and the horizontal line. Lumbar Lordosis was evaluated by the four-line method cobb angle. Measurements of Lumbar Lordosis and Sacral Slope were performed as shown in Figure 1. Participants’ lumbar and paravertebral muscles were scanned using magnetic resonance imaging to obtain cross-sectional images of intervertebral disc midpoints at levels of L1-L2, L2-L3, L3-L4, L4-L5, and L5-S1. After obtaining cross-sectional images of paravertebral muscles, a researcher traced the contours of paravertebral muscles on the screen (Figure 2). Based on profiles of paravertebral muscles, the Image J 1.53 software program (United States National Institutes of Health, Bethesda, Maryland) was used to calculate their cross-sectional areas. The researcher who performed this assay was blinded to the pain site and to other related information of participants (Tsai et al., 2021).

### Evaluation of Pain Intensity and Disability

The visual analog scale was used to evaluate pain intensities of participants. The higher the value, the higher the pain intensity. Disability levels of participants were assessed using the Oswestry Disability Index (ODI) scale. The ODI scale consists of 10 questions, each with a score between 0 and 5. The higher the ODI score, the higher the degree of disability.

### Statistical Analysis

Statistical analyses were performed using SPSS version 26.0 (IBM, Armonk, New York, United States). Descriptive statistics were performed using basic information of the participants, including their age, height, weight, and disease-related information, as well as their VAS scores, ODI scores, disease duration, cross-sectional areas, Lumbar Lordosis, and Sacral Slope. The Shapiro-Wilk test was used to assess data distribution. Paired Student’s t-test was used to compare muscle tone, stiffness, and cross-sectional areas of paravertebral muscles on painful and non-painful sides. The Pearson correlation test (normal distribution) or Spearman correlation test (non-normal distribution) were used to analyze correlations between VAS scores, ODI scores, disease duration, cross-sectional areas (painful side), Lumbar Lordosis, Sacral Slope and muscle tone as well as stiffness of paravertebral muscles on the painful side. Statistical significance was set at *p* ≤ .05.

## Results

### Study Population

Sixty (30 male and 30 female) elderly participants with unilateral CLBP were recruited in this study. Every participant completed the entire trial protocol. The basic information and disease-related details of these participants are shown in Table 1.

**TABLE 1:** Baseline condition of subjects **(*n* = 60)**.

Items	Information
Number of patients	60
Age (years)	65.03 ± 4.09
Gender (male/female)	30/30
BMI	24.48 ± 2.58
Duration of disease (month)	52.73 ± 60.19
Pain location (left/right)	25/35
VAS	4.20 ± 1.67
ODI	36.86 ± 6.16
Lumbar Lordosis (°)	38.26 ± 13.24
Sacral Slope (°)	32.43 ± 10.20

Values are mean ± standard deviation or n. BMI, body mass index.

VAS, visual analogue scale; ODI, the oswestry disability index.

### Statistical Results of the Cross-Sectional Areas of Paravertebral Muscles

The cross-sectional areas of paravertebral muscles on the painful side at L3 level were significantly smaller than those on the non-painful side (*p* < .05). However, there were no significant differences in cross-sectional areas of paravertebral muscles (painful and non-painful side) at other levels (*p* > .05). Table 2 shows the cross-sectional areas of paravertebral muscles of 60 participants.

**TABLE 2:** The cross-sectional areas of paravertebral muscles and statistical analysis results of paired student’s t-test **(*n* = 60)**.

Lumbar segmental	Non-painful side	Painful side	*p* Value
L1	20.12 ± 4.19	19.85 ± 4.05	.134
L2	20.32 ± 4.18	20.18 ± 3.94	.391
L3	21.29 ± 4.39	20.73 ± 3.90	.023^a^
L4	20.84 ± 3.60	20.39 ± 4.41	.100
L5	18.11 ± 3.65	17.81 ± 3.97	.176

Values are mean ± standard deviation.

a Indicates *p* value < .05.

### Biomechanical Parameters of Paravertebral Muscles

**FIGURE 3:**
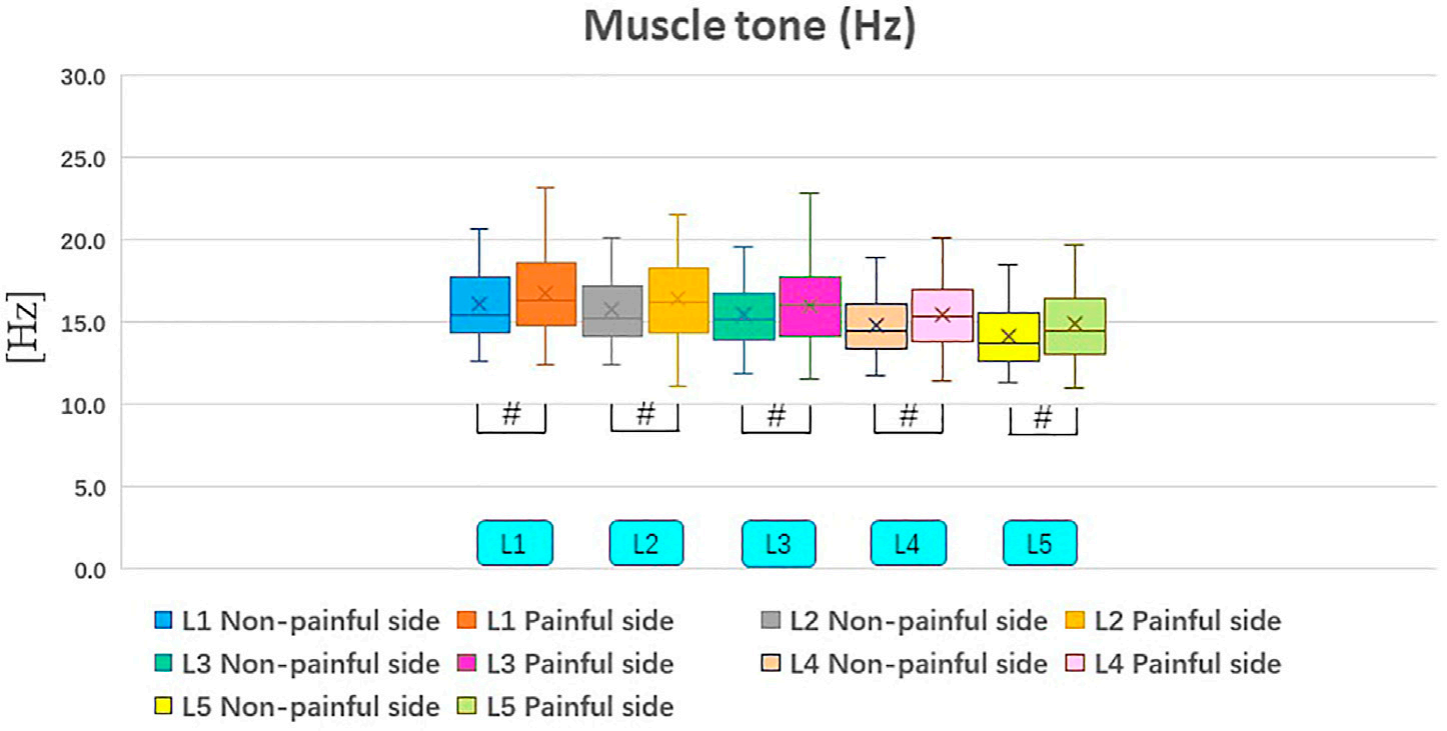
Non-painful and painful side differences in muscle tone. ^#^Indicates *p* value < .01.

**FIGURE 4:**
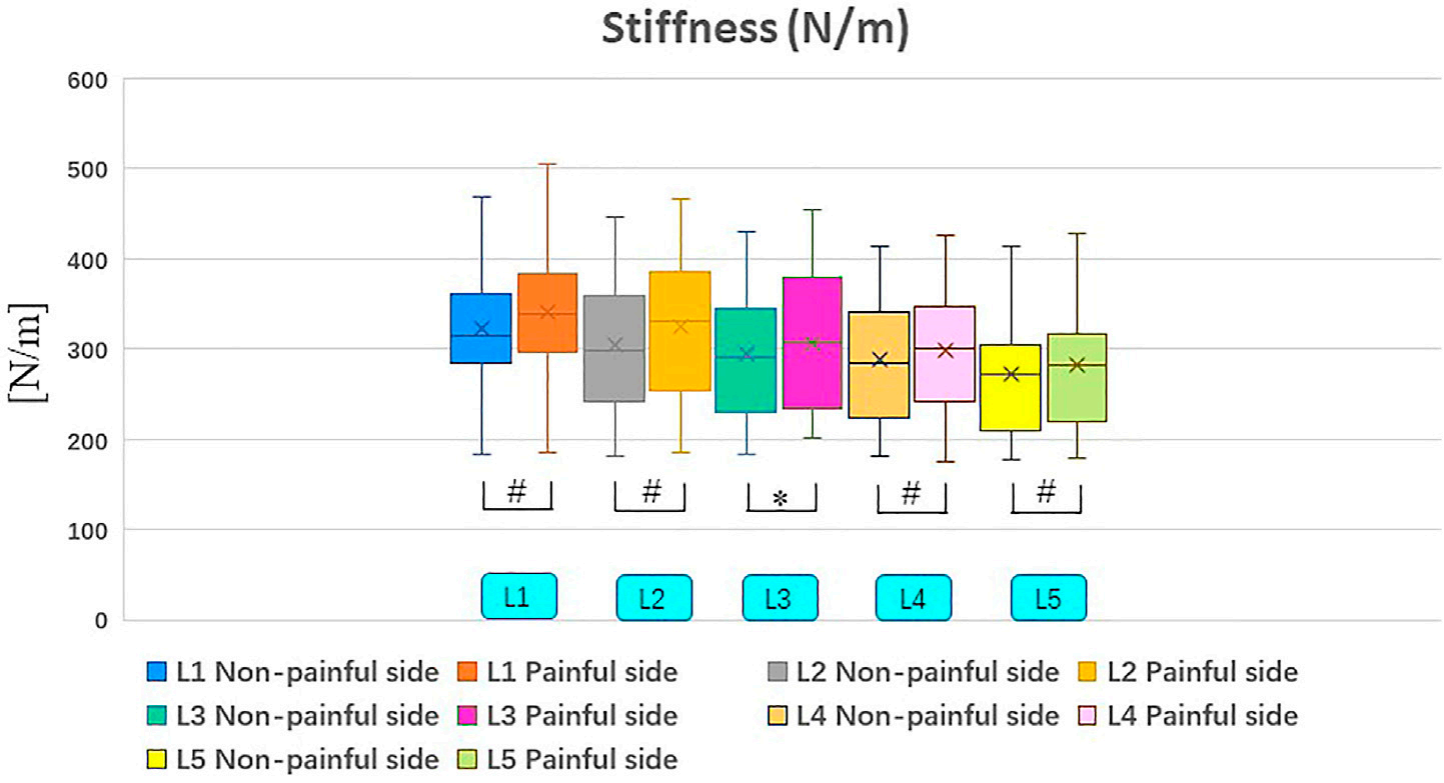
Non-painful and painful side differences in stiffness. ^#^Indicates *p* value < .01; *Indicates *p* value < .05.

Muscle tone on the painful side were significantly higher than those on the non-painful side (Table 3; Figures 3, 4). It was revealed that stiffness of paravertebral muscles on the painful side were significantly higher than on the non-painful side (Table 3; Figures 3, 4). There were positive correlations between biomechanical properties of paravertebral muscles on the painful side and VAS as well as ODI scores. However, there were no correlations with regards to disease duration, cross-sectional areas (painful side), Lumbar Lordosis, and Sacral Slope. VAS scores indicated that muscle tone were positively correlated with painful side paravertebral muscles. ODI scores were also positively correlated with muscle tone of paravertebral muscles on the painful side. Moreover, they were positively correlated with stiffness of paravertebral muscles on the painful side. There were no significant correlations (*p* > .05) between muscle tone and stiffness of paravertebral muscles on the painful side and disease duration, cross-sectional areas (painful side), Lumbar Lordosis, or Sacral Slope (Table 4).

**TABLE 3:** Biomechanical parameters of paravertebral muscles and statistical analysis results of paired student’s t-test **(*n* = 60)**.

Parameters	Levels		Non-painful side	Painful side	*p* Value
Muscle tone (Hz)	L1	Mean ± SD	16.09 ± 2.17	16.79 ± 2.54	<.001^a^
		Range	12.60–22.70	12.40–23.10	
	L2	Mean ± SD	15.77 ± 2.06	16.39 ± 2.58	.009^a^
		Range	12.40–22.10	11.10–24.40	
	L3	Mean ± SD	15.47 ± 2.06	15.99 ± 2.41	<.001^a^
		Range	11.90–22.60	11.50–22.80	
	L4	Mean ± SD	14.81 ± 1.95	15.49 ± 2.40	<.001^a^
		Range	11.80–20.70	11.50–23.10	
	L5	Mean ± SD	14.14 ± 2.03	14.86 ± 2.46	<.001^a^
		Range	11.30–21.20	11.00–22.60	
Stiffness (N/m)	L1	Mean ± SD	322.35 ± 78.11	341.31 ± 79.61	<.001^a^
		Range	183–507	185–518	
	L2	Mean ± SD	305.43 ± 74.02	324.11 ± 76.48	<.001^a^
		Range	182–447	185–466	
	L3	Mean ± SD	294.83 ± 71.03	307.51 ± 74.66	.012^b^
		Range	183–429	202–455	
	L4	Mean ± SD	287.70 ± 70.48	298.33 ± 72.15	.001^a^
		Range	181–413	176–427	
	L5	Mean ± SD	272.65 ± 67.45	282.53 ± 68.95	.001^a^
		Range	177–414	180–428	

SD: standard deviation. The *p* values were derived from the paired-sample *t*-test on the painful and non-painful side.

a Indicates *p* value < .01.

b Indicates *p* value < .05.

**TABLE 4:** Statistical analysis results of correlation test.

Factors	Levels	Muscle tone		Stiffness	
		*r*	*p* Value	*r*	*p* Value
VAS	L1	.323	.012^a^	.286	.027^a^
	L2	.296	.022^a^	.262	.043^a^
	L3	.287	.026^a^	.271	.036^a^
	L4	.300	.020^a^	.311	.015^a^
	L5	.305	.018^a^	.316	.014^a^
ODI	L1	.413	.001^b^	.339	.008^b^
	L2	.397	.002^b^	.352	.006^b^
	L3	.429	.001^b^	.411	.001^b^
	L4	.517	<.001^b^	.391	.002^b^
	L5	.490	<.001^b^	.408	.001^b^
Duration of disease	L1	−.101	.441	.041	.758
	L2	−.109	.406	−.138	.292
	L3	−.109	.409	−.079	.549
	L4	−.052	.694	−.068	.608
	L5	−.117	.374	−.096	.468
Cross-sectional areas (painful side)	L1	.117	.373	.064	.625
	L2	−.021	.872	.057	.665
	L3	.016	.904	.138	.292
	L4	.104	.429	.133	.312
	L5	−.059	.653	−.042	.749
Lumbar Lordosis	L1	−.132	.313	−.028	.831
	L2	−.157	.230	−.042	.749
	L3	−.032	.806	−.051	.692
	L4	−.135	.305	−.071	.590
	L5	−.113	.391	−.099	.451
Sacral Slope	L1	−.026	.845	.011	.931
	L2	−.162	.218	−.049	.713
	L3	−.044	.739	−.097	.462
	L4	−.006	.965	.010	.939
	L5	.001	.993	.023	.863

a Indicates *p* value < .05.

b Indicates *p* value < .01.

## Discussion

Chronic low back pain (CLBP) is caused by multiple factors. However, its pathogenesis has not been clearly established. Various hypotheses have been proposed to explain its mechanisms, including suggestions that CLBP originates from the nerves, lumbar intervertebral discs, lumbar joints, and muscles among others (Morlion, 2013). Studies also suggest that paravertebral muscles are involved in the occurrence and development of CLBP because they play an essential role in maintaining spinal stability (Hides et al., 1996; Sions et al., 2017). Elucidation of the characteristics of trunk muscles of CLBP patients can provide important information for clinical diagnosis and treatment (Sions et al., 2017). Our hypothesis that biomechanical properties of paravertebral muscles are asymmetric between the painful and non-painful sides, and that this is linked to CLBP severity, was supported in this study. However, it was unclear whether changes in biomechanical properties of paravertebral muscles occurred before or after CLBP. Overall, studies support CLBP-induced changes in paravertebral muscles.

According to some studies, morphologies of paravertebral muscles change after CLBP (Hides et al., 1994; Mannion et al., 2000). Other studies have found significant variations in cross-sectional areas of paravertebral muscles between symptomatic and asymptomatic sides of CLBP patients (Hides et al., 1994; Hides et al., 1996), which could be associated with CLBP onset (Hides et al., 2008). The decrease in cross-sectional areas of unilateral paravertebral muscles could be due to disuse atrophy or compensatory hypertrophy of contralateral paravertebral muscles (Dangaria and Naesh, 1998). Pain and an inactive lifestyle may cause paraspinal muscle atrophy, which is significantly associated with pain (Mattila et al., 1986; Danneels et al., 2000; Barker et al., 2004). Masaki et al. (2017) evaluated the relationship between CLBP and muscle stiffness using multiple regression analysis. They found that stiffness of multifidus muscles was an independent influencing factor of low back pain, therefore, they concluded that CLBP-induced muscle spasms was the possible cause for the increase in multifidus muscle stiffness (Masaki et al., 2017). Sherman (1985) investigated the relationship between CLBP and muscle contraction and found that there was a significant positive correlation between pain intensity and muscle contraction. Furthermore, compared to healthy people, CLBP patients exhibit lower muscle strength, endurance, and anti-fatigue abilities (Nicolaisen and Jørgensen, 1985; Roy et al., 1989). According to histological examinations, proportions of type I muscle fibers in muscles of CLBP patients were lower than in asymptomatic patients, the proportions of type II muscle fibers were higher, and anti-fatigue abilities of type II muscle fibers were weaker than those of type I muscle fibers (Mannion et al., 1997). Moreover, muscle stiffness can be caused by a lack of contractile activities when muscles are in a contracted state for extended periods (Park et al., 2017). Pain, among other factors, may cause the paravertebral muscles on the painful side to remain in a contracted state for a long time. Muscle mobility is normally reduced in a contracted state, resulting in changes in transverse bridges of muscle fibers and elimination of sarcomeres (Brauer and Barker, 2003). This could be one of the causes of the increase in muscle tone and stiffness. Moreover, in scoliosis patients, the side of paravertebral muscles with reduced muscle activities exhibited low proportions of type I muscle fibers and high proportions of type II muscle fibers (Mannion et al., 1998). These studies indicate that decreasing muscular activities are correlated with changes in muscular fibers, which could explain the differences in biomechanical properties of paravertebral muscles.

Previous morphological and histological studies of paravertebral muscles in CLBP patients have revealed, to a certain extent, the mechanism of changes in biomechanical properties of paravertebral muscles in patients with unilateral CLBP. The asymmetry of biomechanical properties of paravertebral muscles on painful and non-painful sides may be attributable to various factors, such as increased pain, decreased muscular activities, decreased muscular anti-fatigue abilities, changes in muscle cross-sectional areas, and higher proportions of type II muscle fibers. Although some researchers have carried out relevant studies, these results have not been confirmed by a large number of clinical trials and molecular biological experiments. We speculate that these factors may be related to the biomechanical properties of the paraspinal muscles. In the future, we hope to conduct more clinical trials and molecular biology experiments to analyze further the causes and specific mechanisms of changes in the biomechanical properties of paravertebral muscles.

We found that there were no significant correlations between disease duration and biomechanical properties of paravertebral muscles, which could be due to significant differences in disease duration among participants. Furthermore, CLBP is a pain syndrome with many causes, and disease durations are not positively correlated with CLBP severity; however, they are affected by various factors, such as pain degree and psychological factors (Chaléat-Valayer et al., 2011). Biomechanical parameters of the spine, such as Lumbar Lordosis, play an essential role in maintaining the stability of the lumbar spine (Ko et al., 2018), indicating that changes in Lumbar Lordosis and Sacral Slope are essential to CLBP occurrence. Accordingly, compared to healthy people, there are significant differences in Lumbar Lordosis and Sacral Slopes of CLBP patients with CLBP (Chaléat-Valayer et al., 2011). In addition, curvatures of the lumbar spine and biomechanical changes in the spine can change the tension and pressure of paravertebral muscles and ligaments, leading to CLBP (Jackson and McManus, 1994). We found that there were no significant correlations between Lumbar Lordosis, Sacral Slope, and biomechanical properties of paravertebral muscles. This may be due to the small sample size and to the fact that lumbar lordosis angles and sacral inclination angles for patients were within a small range. In addition, Lumbar Lordosis and Sacral Slope were affected by many factors, such as degree of pain and muscle strength.

Studies reported that cross-sectional areas of bilateral paravertebral muscles in patients with unilateral chronic low back pain are asymmetric (Chon et al., 2017). The asymmetry of cross-sectional areas of paravertebral muscles may be the cause of changes in biomechanical properties. We measured the cross-sectional areas of paravertebral muscles in patients with unilateral chronic low back pain and found that cross-sectional areas of paravertebral muscles on the painful side at L3 level were smaller than those on the non-painful side. However, this may be due to the small number of samples included in the study, and the duration of the disease in these patients was concentrated in a small range. At other levels of the lumbar spine, there were no significant changes in cross-sectional areas of paravertebral muscles or in asymmetry. However, from the measurement results, the cross-sectional area of the paravertebral muscles on the painful side and the non-painful side tends to change. Presently, influencing factors of paravertebral muscle biomechanical asymmetry in elderly patients with unilateral CLBP are unclear. However, based on previous studies involving patients with unilateral CLBP and the findings of this study, it is notable that there were significant differences between painful and non-painful paravertebral muscles, including abnormally elevated muscle tone and stiffness of the paravertebral muscles, as well as a reduction in the cross-sectional area of the paravertebral muscles, and these differences may be of the underlying pathological mechanisms of CLBP. Furthermore, these differences can be combined with other asymmetry variations in paravertebral muscles to further explore the pathogenesis of CLBP.

Besides providing a foundation for evaluating the clinical characteristics of CLBP, our findings may be provide a potential reference for assessing the effectiveness of clinical interventions. At present, many clinical interventions are used to treat CLBP, such as dry needling, myofascial release, etc. (Arguisuelas et al., 2017; Tüzün et al., 2017). However, the evaluation of clinical efficacy is mainly focused on the results reported by patients actively, and the results of subjective reports may lack objectivity. In addition, the biomechanical properties of paravertebral muscles were also used for diagnosis and evaluation of efficacy, but most of them were evaluated by manual palpation (Abbott et al., 2009; Masaki et al., 2017). The reliability of manual palpation in the clinical application was doubted by researchers (Seffinger et al., 2004; Jonsson and Rasmussen-Barr, 2018). Given that the tone and stiffness of the paravertebral muscles in patients with CLBP are higher than those of healthy people, a future study can evaluate whether clinical interventions can reduce these abnormally increased muscle tone and stiffness. Although determination of the root cause of asymmetry of biomechanical properties between painful and non-painful sides is challenging, this study forms the basis for evaluation of CLBP. Future studies should use large sample sizes to assess the specific mechanisms involved in changes in biomechanical characteristics.

### Limitations

This cross-sectional study has several limitations. First, with a small sample size, this study provides a preliminary analysis of biomechanical properties of paravertebral muscles in elderly patients with unilateral CLBP. Second, the results may be affected by disease characteristics of patients, such as disease duration, pain intensity, and degree of daily activities. Third, we did not control the specific pain areas of patients with unilateral chronic low back pain, such as pain in the upper, middle and lower lumbar spines. The included patients may have some bias. Fourth, MRI images are subjected to an increasing shrinking factor the further the distance from the center of the field of view. Shrinking factors at the key points used in this study could not be determined as CT scan images were not available for grometrical comparison. In future studies, these influencing factors should be strictly controlled to reduce the possibility of bias, while further increasing the sample sizes and exploring other new discoveries.

## Conclusion

Biomechanical properties of paravertebral muscles in elderly patients with unilateral CLBP are asymmetrical, and muscle tone as well as stiffness of paravertebral muscles on the painful side are significantly higher than those on the non-painful side. Besides, asymmetric biomechanical properties of paravertebral muscles are associated with CLBP severity.

## Data Availability

The original contributions presented in the study are included in the article/Supplementary Material, further inquiries can be directed to the corresponding author.
